# Insulin-Mimetic Action of Rhoifolin and Cosmosiin Isolated from *Citrus grandis* (L.) Osbeck Leaves: Enhanced Adiponectin Secretion and Insulin Receptor Phosphorylation in 3T3-L1 Cells

**DOI:** 10.1093/ecam/nep204

**Published:** 2011-03-10

**Authors:** Yerra Koteswara Rao, Meng-Jen Lee, Keru Chen, Yi-Ching Lee, Wen-Shi Wu, Yew-Min Tzeng

**Affiliations:** ^1^Institute of Biochemical Sciences and Technology, Chaoyang University of Technology, 168 Gofeng E Road, Wufeng 41349, Taiwan; ^2^Department of Horticulture and Biotechnology, Chinese Culture University, Taipei, Taiwan

## Abstract

*Citrus grandis* (L.) Osbeck (*red wendun*) leaves have been used in traditional Chinese medicine to treat several illnesses including diabetes. However, there is no scientific evidence supporting these actions and its active compounds. Two flavone glycosides, rhoifolin and cosmosiin were isolated for the first time from *red wendun* leaves and, identified these leaves are rich source for rhoifolin (1.1%, w/w). In differentiated 3T3-L1 adipocytes, rhoifolin and cosmosiin showed dose-dependent response in concentration range of o.oo1–5 **μ**M and 1–20 **μ**M, respectively, in biological studies beneficial to diabetes. Particularly, rhoifolin and cosmosiin at 0.5 and 20 **μ**M, respectively showed nearly similar response to that 10 nM of insulin, on adiponectin secretion level. Furthermore, 5 **μ**M of rhoifolin and 20 **μ**M of cosmosiin showed equal potential with 10 nM of insulin to increase the phosphorylation of insulin receptor-**β**, in addition to their positive effect on GLUT4 translocation. These findings indicate that rhoifolin and cosmosiin from *red wendun* leaves may be beneficial for diabetic complications through their enhanced adiponectin secretion, tyrosine phosphorylation of insulin receptor-**β** and GLUT4 translocation.

## 1. Introduction

The worldwide prevalence of type 2 diabetes is increasing at a staggering rate. It has been estimated that by the end of this decade, the number of people having type 2 diabetes will grow to in excess of 320 million [1]. Type 2 diabetes is characterized by an insensitivity of peripheral tissues to the action of insulin, termed insulin resistance. Binding of insulin to its specific cell surface receptor induces receptor autophosphorylation that triggers the activation of two main intracellular signaling pathways: the phosphatidyl-inositol-3-kinase (PI_3_K) pathway involved in glucose transport and glycogen synthesis, and the extracellular regulated kinase (ERK) pathway involved in gene expression and cell proliferation mechanisms. Resistance to insulin can appear at various levels of these signaling pathways [2]. On the other hand, adiponectin is an important endocrine secreted cytokine which affect insulin sensitivity through modulation of insulin signaling pathway [3]. The functions of adiponectin have been the focus of recent attention because accumulating evidence has shown that there are close relationships between circulating adiponectin levels and a variety of lifestyle-related diseases, including obesity, coronary artery disease, type 2 diabetes and metabolic syndrome, and adiponectin exhibits insulin-sensitizing, anti-diabetic and anti-atherogenic activities [4]. Collectively, it is reasonable to consider that adiponectin represents a useful target for treating or preventing insulin resistance, type 2 diabetes. Current pharmacological approach using thiazolidinediones (TZD) such as pioglitazone (PGZ) and rosiglitazone in the treatment of type 2 diabetes have undesirable side effects including weight gain [5], fluid retention, the worsening of coronary heart disease [6], and an increased risk of myocardial infarctions [7]. With these points in mind, the search for alternative therapy to combat type 2 diabetes is warranted.

In recent years, much attention has been paid to the discovery of natural products that may be beneficial in reducing the risks of diabetes, because these are usually considered to be less toxic, with fewer side effects than synthetic drugs [8–14]. In this connection, with the distinctive medical opinions the traditional Chinese herbs and herbal formulae performed a good clinical practice and showing a bright future in the therapy of diabetes mellitus and its complications [15]. *Citrus grandis* (L.) Osbeck of the family Rutaceae is widely cultivated in subtropical areas of Asia. *Red wendun* is the local name for *C. grandis* Osbeck in the Taiwan. The essential oil of this species is used as the major ingredients of flavor, the leaves are used as a food flavoring, and dried leaves are brewed in water as a drink [16]. *Red wendun* leaves have long been used as herbal remedies in traditional Chinese medicine to promote blood circulation and remove blood stasis in diseases caused by blood stagnation [17]. For these reasons, *red wendun* is a traditional Chinese anti-diabetic medicine. However, there is no report on the identities and their mechanisms underlying the anti-diabetic action of constituents from *red wendun* leaves. Therefore, in the present study we used differentiated 3T3-L1 adipocytes to identify potential agents which enhanced adiponectin secretion, tyrosine phosphorylation of the insulin receptor (IR)-*β* subunit, as well as glucose transporter4 (GLUT4) translocation with insulin-mimetic activity. Our finding will significantly contribute to the growing knowledge about *red wendun* leaves for their application in the treatment of diabetic disease.

## 2. Methods

### 2.1. General

All the materials were obtained commercially and used without further purification. NMR spectra were measured on Varian Unity Inova-600 VXR-300/51 spectrometer, using TMS as an internal standard. Silica gel for column chromatography (CC), (0.063–0.200 mm), was a product of Merck Company. TLC was performed on Merck TLC plates (0.23 mm thickness), with compounds visualized by spraying with 8% (v/v) H_2_SO_4_ in ethanol (EtOH) and then heating on a hot plate.

Dexamethasone (DEX), 3-isobutyl-1-methylxanthine (IBMX), insulin (I) and phenylmethylsulfonylfluoride (PMSF) were purchased from Sigma Aldrich Chemical (St. Louis, MO, USA). Dulbecco's Modified Eagle's Medium (DMEM), fetal bovine serum (FBS), and antibiotic mixture (penicillin-streptomycin) were purchased from the Invitrogen Co. (Carlsbad, CA, USA). Polyvinyldifluoride (PVDF) membrane for western blotting was obtained from Millipore (Bedford, MA, USA). Primary antibodies anti-GLUT4, anti-insulin receptor *β* and anti-adiponectin were obtained from Santa Cruz Biotechnology (Santa Cruz, CA, USA). The antibody anti-rabbit IgG conjugated with horseradish peroxidase (HRP) were purchased from the Amersham-Pharmacia Biotech (Arlington Heights, IL, USA). All other chemicals were reagent grade or higher and obtained from commercial sources.

### 2.2. Plant Material, Extraction, and Isolation

*Citrus grandis* (L.) Osbeck leaves were collected from the Madau, Tainan County, Taiwan, in October 2008. The species was identified by one of the author Dr. Yew-Min Tzeng at Natural Products & Bioprocess Laboratory, Chaoyang University of Technology. A voucher specimen (YMT 8001) was deposited in the Herbarium of the Institute of Biochemical Sciences and Technology, Chaoyang University of Technology, Taiwan, ROC.

**Figure 1:**
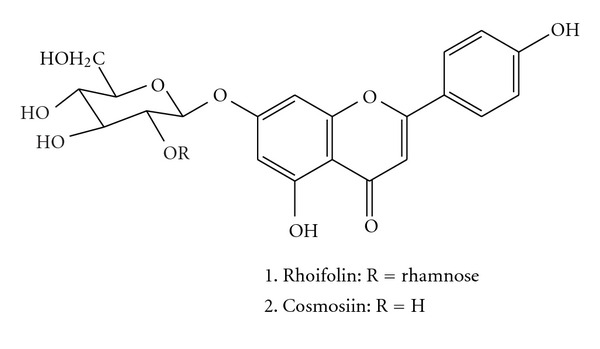
Chemical structures of rhoifolin (**1**) and cosmosiin (**2**) isolated from *C. grandis* leaves.

The air-dried leaves (600 g) of *C. grandis* were extracted with methanol (61 × 4) under reflux. After exhaustive extraction, the combined extracts were concentrated under reduced pressure to give dark brown syrup of *∼*37 g (6.1% based on the dry weight of leaves). The crude extract was then suspended in H_2_O, defatted with *n*-hexane, and then partitioned with chloroform and *n*-butanol successively. The concentrated *n*-butanol layer (15 g) was subjected to silica gel CC and eluted with increasing polarity using mixtures of CHCl_3_/MeOH. Following the TLC analysis, eluates of similar profiles were combined to give five fractions (F1–F5). Fraction F2 (1.2 g) was rechromatographed over a silica gel column using CHCl_3_/MeOH (95 : 5 and 90 : 10) to afford compound **2** as a yellow solid, identified as cosmosiin or apigenin-7-*O*-*β*-glucoside (125 mg, 0.021%). Fraction F3 (9.3 g) was purified further by chromatography on silica gel with CHCl_3_/MeOH, resulting in compound **1** as yellow amorphous powder, identified as rhoifolin or apigenin-7-*O*-*β*-neohesperidoside (6.7 g, 1.1%). The structures of rhoifolin and cosmosiin (Figure 1) were established by comparison of their NMR spectral data with those reported in the literature.

#### 2.2.1. Rhoifolin

Yellow amorphous powder; UV (MeOH) *λ*_max_ (log *ε*): 268 (4.32), 335 (4.1); ^1^H NMR (DMSO-*d*_6_): *δ* 7.95 (2H, d, *J* = 8.5 Hz, H-2′, 6′), 6.95 (2H, d, *J* = 8.5 Hz, H-3′, 5′), 6.87 (1H, s, H-3), 6.79 (1H, d, *J* = 1.5 Hz, H-8), 6.37 (1H, d, *J* = 1.5 Hz, H-6), 5.23 (1H, d, *J* = 7.5 Hz, H-1′′), 5.13 (1H, brs, rham-1), 3.20–3.88 (sugar protons), 1.20 (1H, d, *J* = 6.0 Hz, rham-6); ^13^C NMR (DMSO-*d*_6_) *δ* 181.9 (C = O), 164.3 (C-2), 162.5 (C-7), 161.4 (C-4′), 161.1 (C-5), 156.9 (C-9), 128.6 (C-2′, 6′), 121.0 (C-1′), 116.0 (C-3′, 5′), 105.4 (C-10), 103.2 (C-3), 100.5 (C-1′′), 99.3 (C-6), 97.8 (C-1′′′), 94.5 (C-8), 77.2 (C-3′′), 77.0 (C-5′′), 76.3 (C-2′′), 71.9 (C-4′′′), 70.5 (C-2′′′), 70.4 (C-3′′′), 69.6 (C-4′′), 68.3 (C-5′′′), 60.5 (C-6′′), 18.1 (C-6′′′); ESI-MS (positive mode) *m/z* 579.1 [M + H]^+^ [18].

#### 2.2.2. Cosmosiin

Yellow solid; UV (MeOH) *λ*_max_ (log *ε*): 266 (4.3), 337 (3.9); ^1^H NMR (DMSO-*d*_6_): *δ* 8.02 (2H, d, *J* = 8.5 Hz, H-2′, 6′), 6.89 (2H, d, *J* = 8.5 Hz, H-3′, 5′), 6.87 (1H, s, H-3), 6.78 (1H, s, H-8), 6.27 (1H, s, H-6), 5.01 (1H, d, *J* = 7.5 Hz, H-1′′), 3.20–3.88 (sugar protons); ^13^C NMR (DMSO-*d*_6_) *δ* 182.1 (C = O), 163.9 (C-2), 162.6 (C-7), 161.1 (C-4′), 160.4 (C-5), 156.0 (C-9), 128.5 (C-2′, 6′), 121.6 (C-1′), 115.8 (C-3′, 5′), 104.6 (C-10), 104.0 (C-3), 102.4 (C-1′′), 98.9 (C-6), 98.1 (C-8), 78.6 (C-3′′), 73.4 (C-5′′), 70.8 (C-2′′), 70.5 (C-4′′), 61.3 (C-6′′); ESI-MS (positive mode) *m/z* 433.1 [M + H]^+^ [19].

### 2.3. T3-L1 Adipocyte Culture

3T3-L1 fibroblasts were purchased from the Bioresource Collection and Research Center (BCRC, Food Industry Research and Development Institute, Taiwan, ROC). The cells were cultured as described earlier [20]. Fibroblasts were grown to confluence in 25 mM glucose DMEM containing 10% new calf serum and penicillin/streptomycin (100 IU and 100 *μ*g mL^*‒*1^, resp.) in a humidified atmosphere containing 5% CO_2_. Differentiation to the adipocyte phenotype was induced by incubating cells in DMEM containing 10% FBS, 0.2 nM insulin, 0.25 mM DEX, and 0.5 mM IBMX for 48 h. Cells were then incubated for an additional 48 h in DMEM containing 10% FBS and 0.2 nM of insulin. After differentiation, adipocytes were maintained in normal culture medium (DMEM, 25 mM glucose and 10% FBS) and was freshly replaced every 48 h. Unless indicated otherwise, cells were used 9 days after differentiation induction when exhibiting more than 90% adipocyte phenotype. Stock solutions of rhoifolin and cosmosiin were prepared in DMSO, and equal volume of DMSO (final concentration <0.1%) was added to control samples.

### 2.4. Cell Viability (MTT Assay)

For cell viability, 3T3-L1 cells were seeded in 24-well plates at density of 4 × 10^4^ cells mL^*‒*1^. When they reached 90% confluence, the cells were treated with different concentrations of rhoifolin and cosmosiin for 24 h. Cell viability was determined using MTT 3-(4,5-dimethylthiazol-2-yl)-2,5-diphenyltetrazolium bromide at 0.5 mg mL^*‒*1^ concentration. Treatments were replicated in four wells and experiments repeated at least three times.

### 2.5. Lactate Dehydrogenase Activity (LDH Assay)

For cell viability, 3T3-L1 cells were seeded in 24-well plates at density of 4 × 10^4^ cells mL^*‒*1^. When they reached 90% confluence, the cells were treated with different concentrations of rhoifolin and cosmosiin for 24 h. After 24 h of culture with tested compounds treatment, LDH assay was performed using LDH cytotoxicity detection kit (Takara, Otsu, Shiga, Japan) according to the manufacturer's instructions. Data are presented as cytotoxicity (%) = (experimental value *‒* low control)/(high control *‒* low control) × 100. High control = maximum release of LDH at cell lysis, low control = medium with cultured cells.

### 2.6. Western Blot

3T3-L1 adipocytes were transferred to a serum-free medium for 3 h after 8 days after the initiation of differentiation, and then incubated with 0–20 *μ*M of rhoifolin and cosmosiin at 37°C in a humidified 5%CO_2_ incubator. The incubation period for adiponectin secretion and tyrosine phosphorylation of IR*β* were 24 h and 20 min, respectively. Cells were collected and lysed in ice-cold lysis buffer (1 mM Tris-HCl, 1 mM EDTA, pH 8.0, 200 mM PMSF, 1 mM aprotinin, 1% protease inhibiter cocktail). Culture medium was concentrated by freeze drying and reconstitution. Protein extractions or concentrated culture medium were separated by sodium dodecyl sulfate-polyacrylamide gel electrophoresis (SDS-PAGE) using a 7.5% PAGE. The proteins in the gel were transferred to a PVDF membrane. The PVDF membrane was blocked with 5% skimmed milk in PBST (0.05% v/v Tween-20 in PBS, pH 7.2) for 1 h. Membranes were incubated with primary antibody at 4°C overnight. Primary antibodies used were: anti-adiponectin (1 : 1000), anti-insulin receptor-*β* and anti-GLUT4 (1 : 200). Specific antibody binding was detected by HRP-conjugated secondary antibodies and visualized using western blot detection system (ECL Plus, Amersham, UK). The band densities were quantified using an image analyzer NIH Image software. All protein quantifications were adjusted for the corresponding *β*-actin level, which was not consistently changed by the different treatment conditions.

### 2.7. GLUT4 Translocation

For GLUT4 translocation studies adipocytes were grown on poly-l-lysine coated cover glass and incubated in the presence or absence of either insulin or compounds (rhoifolin and cosmosiin) at 37°C for 20 min. The cells were fixed with 4% paraformaldehyde, blocked with 1% horse serum for 1 h, followed by incubation with two primary antibodies (GLUT4/Na^+^-K^+^ ATPase (Upstate)) in PBST (0.1% Tween-20) at 4°C overnight. After PBS wash, the cells were treated with secondary antibodies (anti-goat IgG conjugated with Alexa Fluor 488 and anti-rabbit IgG conjugated with cy3). The stained cells were visualized and photographed using an Axioscope fluorescent microscope (Zeiss, Oberkochen, Germany).

### 2.8. Statistical Analysis

All values were expressed as means ± SD and one-way ANOVA was used to determine significance of treatment effects at each time point. Differences among treatment means were determined by Tukey's test and *P*-values were considered significant at *P < .05, **P < .01.

## 3. Results

### 3.1. Tested Compounds Rhoifolin and Cosmosiin Are Not Cytotoxic

**Figure 2:**
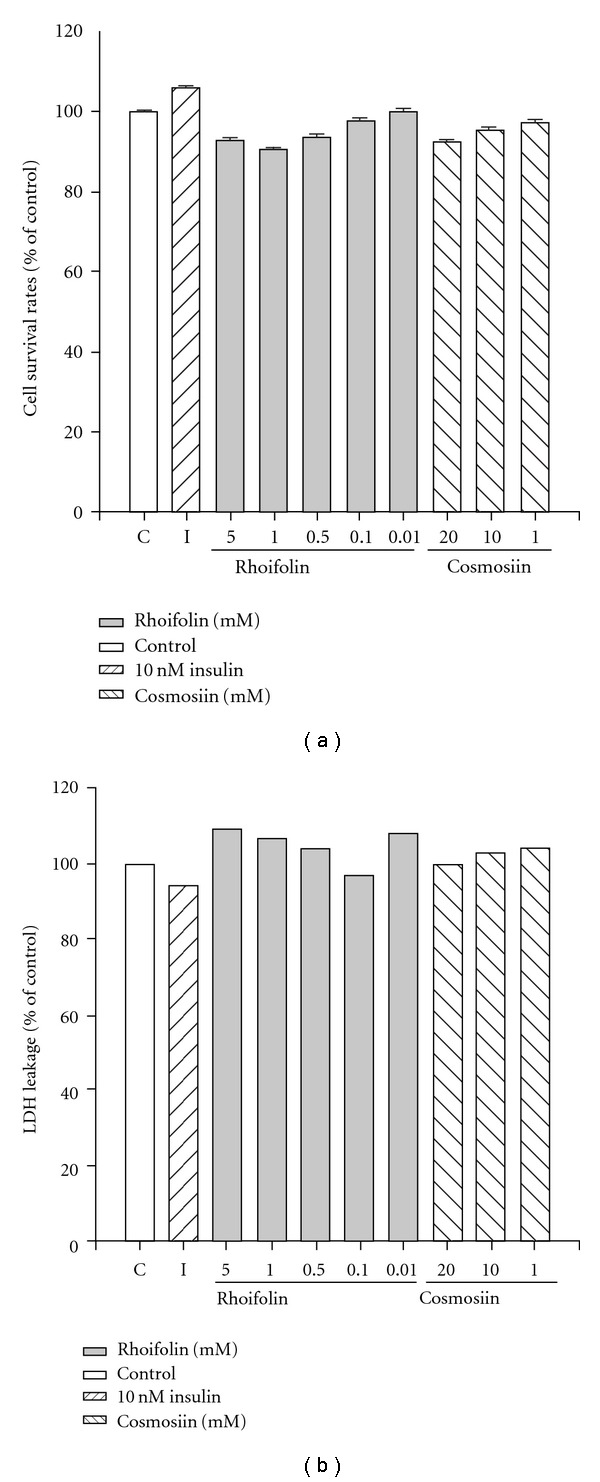
Cell viability analysed by MTT and LDH release assays after 24 h of addition of tested compound or insulin in differentiated 3T3-L1 cells. Differentiation of 3T3-L1 cells was initiated after confluence for 2 days with IBMX and dexmethasone, and compounds were added 9 days after differentiation for 24 h.

We first examined the safety of rhoifolin and cosmosiin on differentiated 3T3-L1 cells viability using the MTT method and LDH release. As shown in Figure 2, compared with untreated control cells, no significant change in viability was observed at various concentrations tested for an incubation period of 24 h (Figure 2). LDH release by rhoifolin and cosmosiin treatment did not increase in differentiated 3T3-L1 cells (Figure 2). We conclude that rhoifolin and cosmosiin did not affect proliferation of differentiated 3T3-L1 cells.

### 3.2. Response of Rhoifolin and Cosmosiin on Adiponectin Secretion

**Figure 3:**
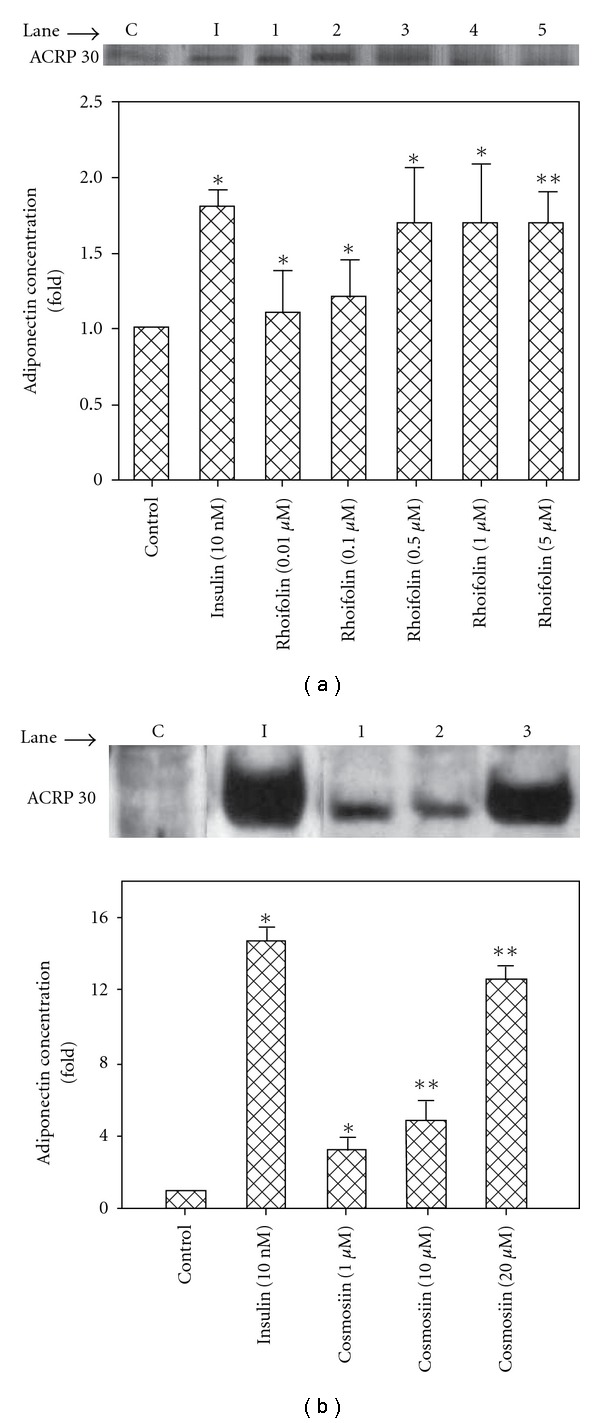
Effect of rhoifolin and cosmosiin on the adiponectin secretion from 3T3-L1 adipocytes. Nine days after the initiation of differentiation, cells were treated with control medium or test compounds for 24 h at 37°C. (a) Control medium (C); 10 nM insulin (I); rhoifolin 0.01 *μ*M (lane 1), 0.1 *μ*M (lane 2), 0.5 *μ*M (lane 3), 1 *μ*M (lane 4), and 5 *μ*M (lane 5). (b) Control medium (C); 10 nM insulin (I); cosmosiin 1 *μ*M (lane 1), 10 *μ*M (lane 2), and 20 *μ*M (lane 3). Secreted adiponectin was quantified by western blotting with antibody. The blots shown are one representative of three independent experiments. Values are means ± S.D. of three experiments and *P < .05; **P < .01 versus control.

Next, to explore the acute response of rhoifolin and cosmosiin on adiponectin secretion, differentiated 3T3-L1 adipocytes were treated with 10 nM of insulin or increasing concentrations of rhoifolin (0.01 *μ*M, 0.1 *μ*M, 0.5 *μ*M, 1 *μ*M, and 5 *μ*M) and cosmosiin (1 *μ*M, 10 *μ*M, and 20 *μ*M) for 24 h and their action on the cells responsiveness to adiponectin release was measured by western blot. As shown in Figure 3, comparisons indicate that secreted adiponectin level in treated sample was significantly different from those of the control. Rhoifolin and cosmosiin showed dose-dependent response in adiponectin secretion levels compared with that of control (Figure 3). The stimulatory response of rhoifolin was maximal at 0.5 *μ*M with increased adiponectin level by 1.7-fold (Figure 3(a), lane 3) and, this response was similar to that of insulin at 10 nM (Figure 3(a), lane I). It should be noted that higher concentrations 1 *μ*M (Figure 3(a), lane 4) and 5 *μ*M (Figure 3(a), lane 5) of rhoifolin had similar response with 0.5 *μ*M of the same compound (Figure 3(a), lane 3). Thus, 0.5 *μ*M of rhoifolin was ideal for maximal adiponectin secretion 3T3-L1 adipocytes. On the other hand, cosmosiin increased adiponectin levels by 2.3 (Figure 3(b), lane 1), 3.8 (Figure 3(b), lane 2), and 11.6 (Figure 3(b), lane 3)-fold at concentration of 1, 10, and 20 *μ*M, respectively, whereas insulin (Figure 3(b), lane I) enhanced the same by 13.7-fold at 10 nM.

### 3.3. Rhoifolin and Cosmosiin Enhance Phosphorylation of Insulin Receptor (pIR)-*β*

**Figure 4:**
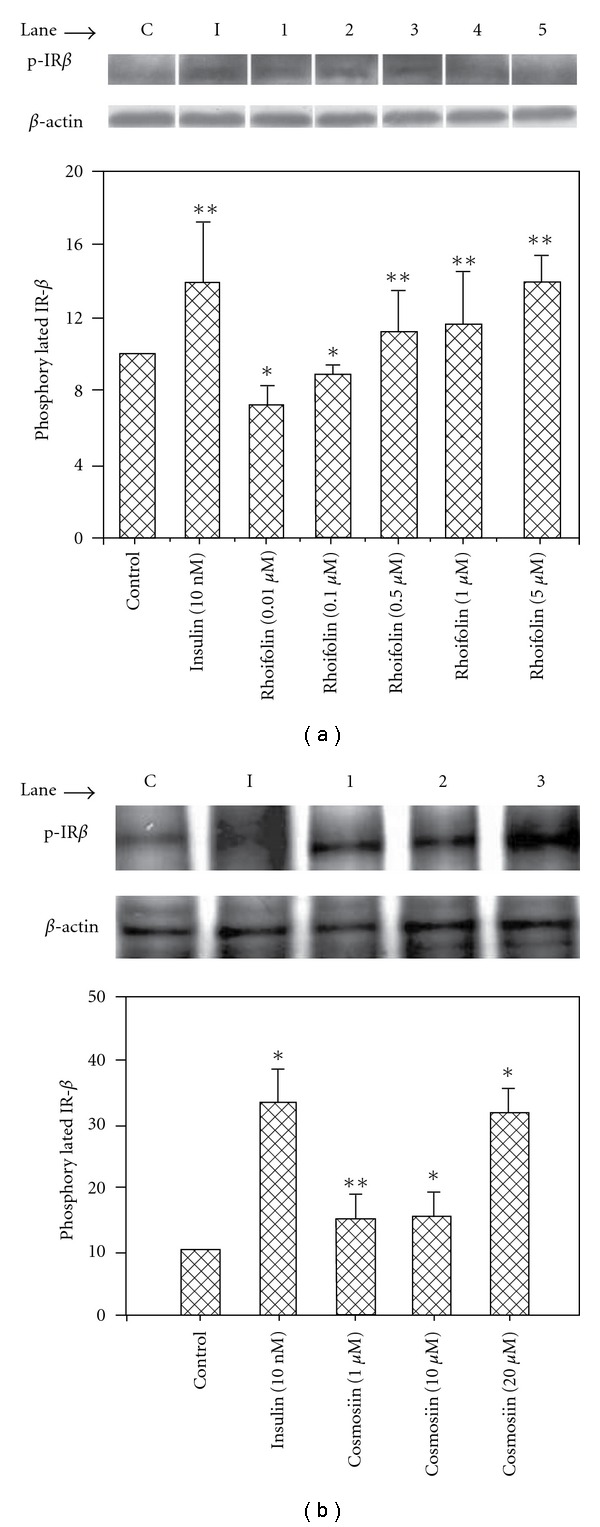
Effect of rhoifolin and cosmosiin on the phosphorylation level of insulin receptor-*β*. Nine days after the initiation of differentiation, cells were treated with control medium or test compounds for 20 min at 37∘C, and then lysates were prepared. (a) Control medium (C); 10 nM insulin (I); rhoifolin 0.01 *μ*M (lane 1), 0.1 *μ*M (lane 2), 0.5 *μ*M (lane 3), 1 *μ*M (lane 4), and 5 *μ*M (lane 5). (b) Control medium (C); 10 nM insulin (I); cosmosiin 1 *μ*M (lane 1), 10 *μ*M (lane 2), and 20 *μ*M (Lane 3). Phosphorylation of IR*β* was assessed by western blot with anti-phospho-IR*β* antibody. Values are means ± SD of three experiments and *P < .05; **P < .01 compared with control.

The levels of tyrosine pIR-*β* in response to 10 nM insulin or different concentrations of rhoifolin and cosmosiin were determined by western blot with anti-pTyr antibody (Figure 4). As shown in Figure 4, lower concentrations of rhoifolin at 0.01 *μ*M (Figure 4(a), lane 1), and 0.1 *μ*M (Figure 4(a), lane 2) had no significant response compared with control (Figure 4(a), lane C) on enhanced pIR-*β* level. However, rhoifolin at concentrations of 0.5 *μ*M (Figure 4(a), lane 3), 1 *μ*M (Figure 4(a), Lane 4), and 5 *μ*M (Figure 4(a), lane 5) showed dose-dependent response and, pIR-*β* levels enhanced by 1.1-, 1.6-, and 3.9-fold, respectively. The response of rhoifolin at 5 *μ*M on enhanced pIR-*β* level was similar to that of insulin at 10 nM (Figure 4(a), lane I). On the other hand, cosmosiin showed a dose-dependent response and, pIR-*β* levels enhanced by 0.5-, 0.6-, and 2.2-fold when the cells were treated with 1 *μ*M (Figure 4(b), lane 1), 10 *μ*M (Figure 4(b), lane 2), and 20 *μ*M (Figure 4(b), lane 3), respectively. It is observed that rhoifolin potentially enhanced pIR-*β* level compared with cosmosiin, in addition, 5 *μ*M of former, and 20 *μ*M of latter showed nearly similar response to that of insulin at 10 nM.

### 3.4. GLUT4 Translocation Was Enhanced by Rhoifolin and Cosmosiin Treatment

**Figure 5:**
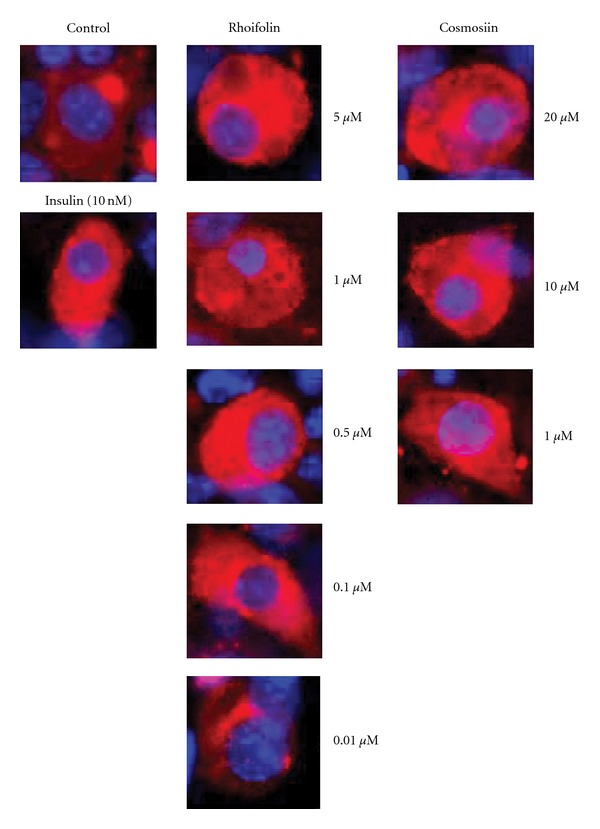
Rhoifolin and cosmosiin stimulated GLUT4 translocation in the 3T3-L1 adipocytes. Cells were starved for 3 h in serum-free medium, then insulin, or different concentrations of rhoifolin and cosmosiin added for 30 min. The subsequent GLUT4 translocation was visualized by fluorescent immunostaining. GLUT4 (red) immunoreactivity was observed near the nucleus (blue) or distributed evenly on the plasma membrane.

We then examined the action of rhoifolin and cosmosiin on the glucose transport system in differentiated 3T3-L1 adipocytes. As shown in Figure 5, the control cells double stained with GLUT4 and nucleus marker DAPI, the GLUT4 immunoreactivity was localized near the nucleus at the Golgi apparatus. However, after stimulation with 10 nM of insulin or various concentrations of rhoifolin (0.5, 1, and 20 *μ*M) and cosmosiin (1, 10, and 20 *μ*M), GLUT4 immunoreactivity moved to the plasma membrane and was not localized near the nucleus (Figure 5). At 0.01 *μ*M, 0.1 *μ*M of rhoifolin GLUT4 translocation was not significant and GLUT immunoreactivity was located near the nucleus.

## 4. Discussion

Although traditional herbal medicines have been practiced for diabetic complications; however, proper mechanism of active compounds of these medicines are not well-understood to support their scientific merit over the existing therapies [21]. This study may provide an initial insight into the possible mechanism of action of *red wendun* leaves as anti-diabetic. In this study, the MeOH extract of *red wendun* leaves was subjected to solvent fractionation and, *n*-butanol fraction was purified using a silica gel chromatography, which led to the isolation of two flavonoids rhoifolin (**1**) and cosmosiin (**2**) (Figure 1). To our knowledge these two flavonoids were isolated for the first time from *red wendun* leaves and, identified these leaves are rich source for the molecule rhoifolin (1.1%, w/w).

Flavonoids are a large group of polyphenolic phytochemicals found in a variety of plant kingdom, medicinal herbs, vegetables and fruits. These natural products are of interest because of their diverse biological activities, such as anti-atherosclerotic, anti-inflammatory, anti-cancer and anti-diabetic actions [22]. Their anti-diabetic role has been indicated in some large, prospective studies. For example, a flavonol diglycoside, kaempferol-3-neohesperidoside has a significant hypoglycemic effect in diabetic rats and stimulate glucose uptake in rat soleus muscle mimicking insulin signaling [23, 24]. Also, genistein and daidzein flavonoids have been reported as insulin-secretagogues since they increased serum insulin secretion stimulated by glucose *in vivo* and *in vitro* [25]. Apigenin, the aglycone of rhoifolin and cosmosiin also reported to have stimulatory effect on glucose uptake [26]. The present compounds rhoifolin and cosmosiin previously reported to have anti-oxidant and immunomodulatory activities [27, 28]. In this study, no appreciable change in cell viability assays (MTT and LDH release) of differentiated 3T3-L1 cells was observed, suggesting that rhoifolin and cosmosiin did not affect cell growth of 3T3-L1 cells (Figure 2).

Adiponectin is one of the most abundant secretary proteins in the adipose tissue of both rodents and humans. Adiponectin is thought to have novel insulin-sensitizing property *in vitro* and *in vivo*, and suggested that it plays a protective role against insulin resistance [4]. In the present study, we observed that an increase in adiponectin levels in differentiated 3T3-L1 adipocytes treated with rhoifolin and cosmosiin after 24 h of incubation (Figure 3). Based on the similarity of cellular effects elicited by the compounds rhoifolin and cosmosiin and, insulin, it is not surprising to find that, like insulin, isolated compounds can stimulate adiponectin release. In relation to the present studies, it must be noted that a polymethoxy flavonoid nobiletin isolated from *Citrus depressa* promoted adiponectin secretion in a concentration-dependent manner in ST-13 cells [29]. A biflavone isoginkgetin isolated from the leaf extracts of *Ginkgo biloba* also reported to enhanced adiponectin production in 3T3-L1 adipocytes [30]. Furthermore, it has been reported that anti-diabetic drug pioglitazone shown to have insulin-like activities through increase adiponectin secretion in 3T3-L1 adipocytes [31]. It is thus possible that rhoifolin and cosmosiin could have in lane effect of these known anti-diabetic compounds in terms of adiponectin secretion. Our study first provides evidence that rhoifolin and cosmosiin elevates the production of adiponectin secretion in 3T3-L1 adipocytes. Future studies will be required to determine the mechanisms by which rhoifolin and cosmosiin increases adiponectin secretion and/or expression.

**Figure 6:**
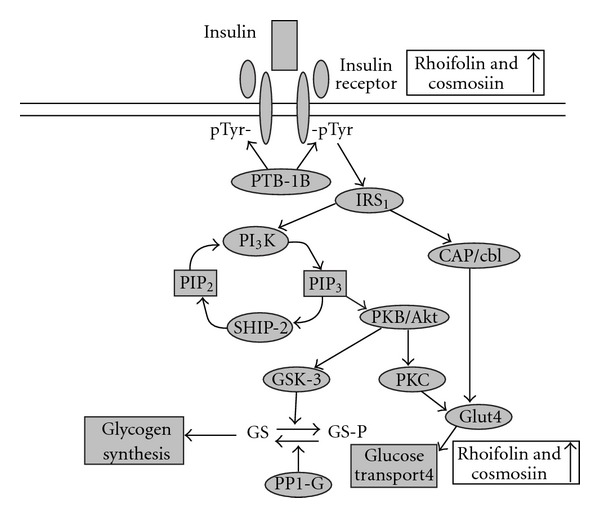
Insulin signaling pathway in adipocytes: showing potential sites of rhoifolin and cosmosiin interactions.

The action of insulin is initiated by its binding to the insulin receptor (IR). This leads to autophosphorylation of the insulin receptor (pIR) and this, in turn, leads to activation of number of proteins including insulin receptor substrate (IRS) and phosphatidylinositol 3-kinase (PI_3_K, see Figure 6). Recently, small-molecule compounds have been discovered that specifically bind to the IR and augment IR-dependent signal transduction cascade, thereby mimicking the action of insulin and could be useful in the treatment of type 2 diabetes [32]. Our results showed that 5 *μ*M of rhoifolin and 20 *μ*M of cosmosiin have nearly similar response to that of insulin at 10 nM on pIR*β* (Figure 4). These findings suggested that the stimulation of adiponectin secretion by these flavones is partly related to the enhanced pIR-*β* and, adipocytes could be target sites to exert their insulin-mimetic activity.

GLUT4 plays an important role in stimulating glucose uptake into muscles and adipocytes. This is achieved by increasing the membrane translocation and activation of GLUT4 in an insulin-dependent or independent manner [33]. Compounds that potentiate GLUT4 translocation to the membrane can be beneficial for the treatment of metabolic syndrome and type 2 diabetes [33]. Extracts from different plants were reported to modulate glucose uptake activity in cultured cells. However, very few studies have been carried out to isolate and characterize the active compound/s from the crude extracts and understand the molecular mechanism by which these compounds affect glucose uptake [34–36]. Our observations revealed activated GLUT-4 translocation to the membrane by insulin in differentiated 3T3-L1 adipocytes (Figure 5). In addition, it is also identified that the varying concentrations of rhoifolin and cosmosiin isolated from *red wendun* leaves facilitated the GLUT4 translocation (Figure 5). The GLUT4 translocation pattern by these flavone glycosides is similar to that of pIR*β*, suggesting that the GLUT4 translocation by these compounds is involved in stimulation of insulin signaling by enhanced pIR-*β* (Figure 6). Although the detailed mechanisms of action need to be investigated further, this is the first study to reveal the potential insulin-mimetic effects of rhoifolin and cosmosiin based on their enhanced adiponectin secretion, pIR-*β* and GLUT4 translocation (Figure 6). Thus, the present results provide a possible explanation for the anti-diabetic effects of *red wendun* leaves. The results of this study showed that rhoifolin was potent than cosmosiin in terms of adiponectin secretion, pIR-*β* and GLUT4 translocation, which might be attributable to the presence of additional rhamnose moiety in rhoifolin.

In conclusion, this study presents for the first time that compounds rhoifolin and cosmosiin from *red wendun* leaves might exert their anti-diabetic effects through enhanced adiponectin secretion, phosphorylation of insulin receptor-*β*, and GLUT4 translocation. The leaves of *red wendun* are a rich source for the molecule rhoifolin (1.1%, w/w). The present compounds may be useful to develop potent lead compounds for the treatment of diabetes through promoting the adiponectin secretion and tyrosine phosphorylation of insulin receptor-*β* and GLUT4 translocation. The critical genes involved in rhoifolin and cosmosiin action may provide novel targets to combat insulin resistance associated diseases.

## References

[1] Ford ES, Li C, Sattar N (2008). Metabolic syndrome and incident diabetes. *Diabetes Care*.

[2] Karpac J, Jasper H (2009). Insulin and JNK: optimizing metabolic homeostasis and lifespan. *Trends in Endocrinology and Metabolism*.

[3] Iannucci CV, Capoccia D, Calabria M, Leonetti F (2007). Metabolic syndrome and adipose tissue: new clinical aspects and therapeutic targets. *Current Pharmaceutical Design*.

[4] Kadowaki T, Yamauchi T, Kubota N (2008). The physiological and pathophysiological role of adiponectin and adiponectin receptors in the peripheral tissues and CNS. *FEBS Letters*.

[5] Boden G, Zhang M (2006). Recent findings concerning thiazolidinediones in the treatment of diabetes. *Expert Opinion on Investigational Drugs*.

[6] Berlie HD, Kalus JS, Jaber LA (2007). Thiazolidinediones and the risk of edema: a meta-analysis. *Diabetes Research and Clinical Practice*.

[7] Nissen SE, Wolski K (2007). Effect of rosiglitazone on the risk of myocardial infarction and death from cardiovascular causes. *The New England Journal of Medicine*.

[8] Jung M, Park M, Lee HC, Kang YH, Kang ES, Kim SK (2006). Antidiabetic agents from medicinal plants. *Current Medicinal Chemistry*.

[9] Samane S, Noël J, Charrouf Z, Amarouch H, Haddad PS (2006). Insulin-sensitizing and anti-proliferative effects of Argania spinosa seed extracts. *Evidence-Based Complementary and Alternative Medicine*.

[10] Klein G, Kim J, Himmeldirk K, Cao Y, Chen X (2007). Antidiabetes and anti-obesity activity of *Lagerstroemia speciosa*. *Evidence-Based Complementary and Alternative Medicine*.

[11] Singh SK, Rai PK, Jaiswal D, Watal G (2008). Evidence-based critical evaluation of glycemic potential of *Cynodon dactylon*. *Evidence-Based Complementary and Alternative Medicine*.

[12] Said O, Fulder S, Khalil K, Azaizeh H, Kassis E, Saad B (2008). Maintaining a physiological blood glucose level with ’glucolevel’, a combination of four anti-diabetes plants used in the traditional Arab herbal medicine. *Evidence-Based Complementary and Alternative Medicine*.

[13] Vosough-Ghanbari S, Rahimi R, Kharabaf S (2008). Effects of *Satureja khuzestanica* on serum glucose, lipids and markers of oxidative stress in patients with type 2 diabetes mellitus: a double-blind randomized controlled trial. *Evidence-Based Complementary and Alternative Medicine*.

[14] Bhat M, Zinjarde SS, Bhargava SY, Kumar AR, Joshi BN Antidiabetic Indian plants: a good source of potent amylase inhibitors.

[15] Wang E, Wylie-Rosett J (2008). Review of selected Chinese herbal medicines in the treatment of type 2 diabetes. *Diabetes Educator*.

[16] Wang Y-C, Chuang Y-C, Hsu H-W (2008). The flavonoid, carotenoid and pectin content in peels of citrus cultivated in Taiwan. *Food Chemistry*.

[17] Li SJ (1980). Self-incompatibility in Matou Wendun (*Citurs Grandis* (L.) Osb.). *Horticultural Science*.

[18] Kaneko T, Sakamoto M, Ohtani K (1995). Secoiridoid and flavonoid glycosides from *Cynodon dactylon*. *Phytochemistry*.

[19] Bennini B, Chulia AJ, Kaouadji M, Thomasson F (1992). Flavonoid glycosides from *Erica cinerea*. *Phytochemistry*.

[20] Frost SC, Lane MD (1985). Evidence for the involvement of vicinal sulfhydryl groups in insulin-activated hexose transport by 3T3-L1 adipocytes. *The Journal of Biological Chemistry*.

[21] Tiwari AK, Rao JM (2002). Diabetes mellitus and multiple therapeutic approaches of phytochemicals: present status and future prospects. *Current Science*.

[22] Nijveldt RJ, Van Nood E, Van Hoorn DEC, Boelens PG, Van Norren K, Van Leeuwen PAM (2001). Flavonoids: a review of probable mechanisms of action and potential applications. *American Journal of Clinical Nutrition*.

[23] Cazarolli LH, Zanatta L, Jorge AP (2006). Follow-up studies on glycosylated flavonoids and their complexes with vanadium: their anti-hyperglycemic potential role in diabetes. *Chemico-Biological Interactions*.

[24] Zanatta L, Rosso Â, Folador P (2008). Insulinomimetic effect of kaempferol 3-neohesperidoside on the rat soleus muscle. *Journal of Natural Products*.

[25] Liu D, Zhen W, Yang Z, Carter JD, Si H, Reynolds KA (2006). Genistein acutely stimulates insulin secretion in pancreatic *β*-cells through a cAMP-dependent protein kinase pathway. *Diabetes*.

[26] Li W, Dai R-J, Yu Y-H (2007). Antihyperglycemic effect of *Cephalotaxus sinensis* leaves and GLUT-4 translocation facilitating activity of its flavonoid constituents. *Biological and Pharmaceutical Bulletin*.

[27] He Z-D, Lau K-M, But PP-H (2003). Antioxidative glycosides from the leaves of *Ligustrum robustum*. *Journal of Natural Products*.

[28] Mikhaeil BR, Badria FA, Maatooq GT, Amer MM (2004). Antioxidant and immunomodulatory constituents of henna leaves. *Zeitschrift für Naturforschung. Section C*.

[29] Kunimasa K, Kuranuki S, Matsuura N (2009). Identification of nobiletin, a polymethoxyflavonoid, as an enhancer of adiponectin secretion. *Bioorganic and Medicinal Chemistry Letters*.

[30] Liu G, Grifman M, Macdonald J, Moller P, Wong-Staal F, Li Q-X (2007). Isoginkgetin enhances adiponectin secretion from differentiated adiposarcoma cells via a novel pathway involving AMP-activated protein kinase. *Journal of Endocrinology*.

[31] Pereira RI, Leitner JW, Erickson C, Draznin B (2008). Pioglitazone acutely stimulates adiponectin secretion from mouse and human adipocytes via activation of the phosphatidylinositol 3′-kinase. *Life Sciences*.

[32] Khan AH, Pessin JE (2002). Insulin regulation of glucose uptake: a complex interplay of intracellular signalling pathways. *Diabetologia*.

[33] Cushman SW, Wardzala LJ (1980). Potential mechanism of insulin action on glucose transport in the isolated rat adipose cell. Apparent translocation of intracellular transport systems to the plasma membrane. *The Journal of Biological Chemistry*.

[34] Wang P-H, Tsai M-J, Hsu C-Y, Wang C-Y, Hsu H-K, Weng C-F (2008). *Toona sinensis* Roem (Meliaceae) leaf extract alleviates hyperglycemia via altering adipose glucose transporter 4. *Food and Chemical Toxicology*.

[35] Purintrapiban J, Suttajit M, Forsberg NE (2006). Differential activation of glucose transport in cultured muscle cells by polyphenolic compounds from *Canna indica* L. root. *Biological and Pharmaceutical Bulletin*.

[36] Vijayakumar MV, Singh S, Chhipa RR, Bhat MK (2005). The hypoglycaemic activity of fenugreek seed extract is mediated through the stimulation of an insulin signalling pathway. *British Journal of Pharmacology*.

